# Horsetail (*Equisetum hyemale*) Extract Accelerates Wound Healing in Diabetic Rats by Modulating IL-10 and MCP-1 Release and Collagen Synthesis

**DOI:** 10.3390/ph16040514

**Published:** 2023-03-30

**Authors:** Hilda Aguayo-Morales, Crystel A. Sierra-Rivera, Jesús A. Claudio-Rizo, Luis E. Cobos-Puc

**Affiliations:** Facultad de Ciencias Químicas, Unidad Saltillo, Universidad Autónoma de Coahuila, Boulevard Venustiano Carranza S/N Esquina con Ing. José Cárdenas Valdés, República Oriente, Saltillo 25290, Mexico

**Keywords:** *Equisetum hyemale*, inflammation, cytokines, phenolic compounds, wound healing, type 2 diabetic rats

## Abstract

Traditionally, *Equisetum hyemale* has been used for wound healing. However, its mechanism of action remains to be elucidated. For this purpose, a 40% ethanolic extract of *E. hyemale* was prepared. Phytochemical screening revealed the presence of minerals, sterols, phenolic acids, flavonols, a lignan, and a phenylpropenoid. The extract reduced the viability of RAW 264.7 cells and skin fibroblasts at all times evaluated. On the third day of treatment, this reduction was 30–40% and 15–40%, respectively. In contrast, the extract increased the proliferation of skin fibroblasts only after 48 h. In addition, the extract increased IL-10 release and inhibited MCP-1 release. However, the extract did not affect both TGF-β1 and TNF-α released by RAW 264.7 cells. The higher release of IL-10 could be related to the up-/downregulation of inflammatory pathways mediated by the extract components associated with their bioactivity. The extract inhibited the growth of *Staphylococcus aureus* and *Escherichia coli*. Topical application of the extract accelerated wound healing in diabetic rats by increasing fibroblast collagen synthesis. These results suggest that *E. hyemale* extract has great potential for use in the treatment of wounds thanks to its phytochemical composition that modulates cytokine secretion, collagen synthesis, and bacterial growth.

## 1. Introduction

Plants of the genus *Equisetum* are used as herbal medicines in various regions of the world. About 20 species are naturally distributed throughout North America (Arctic Circle, Canada, United States, and Mexico), Mesoamerica, South America, Europe, and Northeast Asia to China, except for Australia and New Zealand [1]. The most common species in Mexico are *E. arvense*, *E. hyemale*, *E. laevigatum*, *E. myriochaetum*, and *E. telmateia* [1], and the most commonly used species is *E. hyemale* (horsetail, cola de caballo, kuture, or carricillo in Spanish) [2]. This plant is commercially available in herbariums and apothecaries and can be found along the banks of rivers and streams, in shallow water where the stem grows. *E. hyemale* is a herb up to two meters tall, with brittle, cylindrical hollow stems, unbranched, dark green, and spaced rings running around the stem, emerging from the joints. The fruits are small cones that are found in the terminal part of the plant [3]. This plant has been used in Mexico since pre-Columbian times as an astringent, anti-diabetic, diuretic, and for the treatment of kidney stones [4]. For these uses, the dry leaves and stems are prepared in an aqueous infusion. However, high concentrations of the raw plant material can cause adverse effects such as cardiac arrhythmias, muscle weakness, and erectile dysfunction, probably owing to its elevated thiaminase levels [2]. However, the fractionation by separation techniques avoids toxic reactions in mice [5]. 

Several extraction techniques have been used to obtain different fractions of *E. hyemale*, including percolation, maceration, and ultrasound, by using solvents such as water, ethanol, methanol, ethyl acetate, dichloromethane, and ether [5,6,7,8,9]. The main disadvantage of the above-mentioned techniques is that it usually takes several hours or even days to extract the bioactive molecules. In contrast, ultrasound-/microwave-assisted extraction is a fast and efficient technique because it facilitates the penetration of the heat generated by the microwave and promotes the formation of ultrasonic cavitation bubbles in the plant matrix, both of which aid in the extraction of phenolic compounds such as phenolic acids and flavonoids [10].

Recently, *E. hyemale* has been used to treat diseases such as hypertension, stroke, hemorrhage, cancer, and inflammatory diseases [2,4,11], but the possible mechanisms of action for each condition are still under investigation. Nevertheless, the potential use of *E. hyemale* in inflammatory diseases must be related to the plant’s phenolic compounds (i.e., flavonoids), which can inhibit lymphocyte activation and the production of interferon-gamma (IFN-γ) and tumor necrosis factor-alpha (TNF-α) [12]. Phenolic compounds such as cinnamic acid, chlorogenic acid, caffeic acid, rutin, caffeoyl tartaric acid, kaempferol, quercetin, luteolin, and flavones are present in the *E. hyemale* plant from different geographical regions [5,7,13]. Several species of *Equisetum* (e.g., *E. pyramidale* and *E. arvense*) have anti-inflammatory [12] and wound-healing properties [6,14]. It seems likely that there is a link between the anti-inflammatory action of *Equisetum* plants and their wound-healing effects, as the wound-healing process begins with the coagulation phase (hemostasis), which prevents blood loss from the body. This is followed by an inflammatory phase in which tissue-damaging agents are eliminated. During this phase, cells such as neutrophils, monocytes, and macrophages release pro- and anti-inflammatory mediators. Subsequently, the fibroblasts and keratinocytes migrate from the skin to the damaged tissue for wound epithelialization (proliferation) and, finally, in the tissue remodeling phase, the scar is formed and organized [15]. On this basis, it is evident that inflammation is critical for wound healing. 

A significant percentage of diabetic patients develop chronic wounds. A chronic wound occurs because the healing process is delayed, representing a high economic cost to healthcare institutions and reducing the quality of life of these patients [16]. The most critical cause of delayed wound healing is bacterial infection due to persistent inflammation. Wound infections (diabetic foot ulcers) are usually caused by bacteria from the skin flora such as *Staphylococcus aureus* (*S. aureus*), *Staphylococcus epidermis* (*S. epidermis*), *Pseudomonas aeruginosa* (*P. aeruginosa*), *Streptococcus* spp., and *Enterococcus* spp., which promote the growth of other pathogenic bacteria such as *Escherichia coli* (*E. coli*) [17,18]. It is generally accepted that plant extracts containing phytochemicals such as polyphenols and/or flavonoids have antibacterial activity. In this sense, the ethanolic extract of the Brazilian *E. hyemale* plant has been shown to inhibit the growth of bacteria such as *S. aureus* and *E. coli* and fungi such as *Candida albicans* [7]. Based on this, the present study aims to identify phytochemical compounds in the ethanolic extract (40% v/v) from the stem of the Mexican horsetail (*E. hyemale*) plant obtained by ultrasound-/microwave-assisted extraction and to analyze whether the extract induces anti-inflammatory and antimicrobial responses and accelerates wound healing in diabetic rats, allowing to identify its mechanism of action.

## 2. Results and Discussion

### 2.1. Chemical Elemental Analysis in the Raw Plant Material of E. hyemale 

The chemical elements present in the raw plant material of *E. hyemale* were analyzed by X-ray fluorescence. Table 1 shows that potassium (K), calcium (Ca), and silicon (Si) are the major chemical elements in the raw material, with traces of chlorine (Cl), sulfur (S), phosphorus (P), iron (Fe), and magnesium (Mg). The total content of these elements is equivalent to 14.3% after adjustment for ash, as determined by TGA. 

The minerals contained in the raw material are essential nutrients for the organism and are involved in several biological functions. For example, iron and magnesium are necessary for the regulation of acute and chronic inflammation and for the prevention of bacterial infections [19,20,21,22]. 

### 2.2. Thermal Analysis of Plant Extract 

Thermogravimetric analysis (TGA) measures the loss of mass of each component of a mixture. It can provide an overview of water release and decomposition of the samples as a function of increasing temperature. Typical applications of TGA include the following: determining the thermal stability of a material; the purity of a mineral, inorganic compound, or organic material; improving product formulation processes; or ensuring product safety. TGA allows us to know the chemical composition and the interactions that the biomolecules of the extract establish to form a structure with amorphous order, and it also allows us to know the organic composition (bioactive molecules such as polyphenols, glycosides, and saponins) in % and the inorganic composition (minerals such as silicon, magnesium, and calcium oxides, mainly) also in %, in the extract of horsetail. 

**Figure 1:**
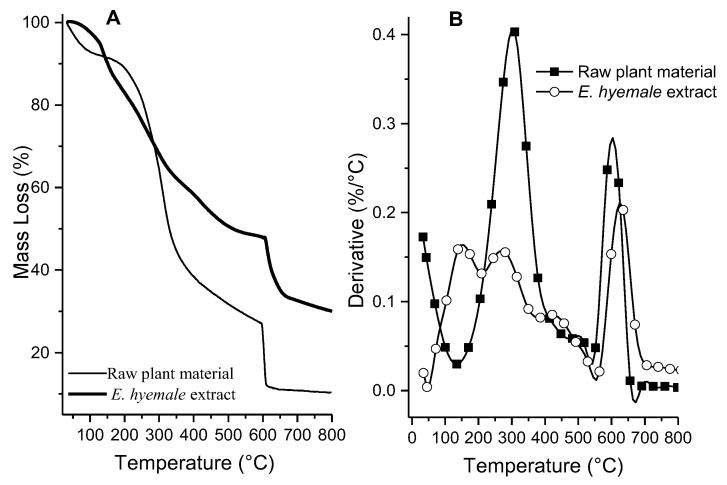
TGA curve (**A**) and first derivative of the TGA thermogram (**B**) for raw plant material and *E. hyemale* extract.

The TGA thermograms show three regions of mass loss: (1) evaporation of water and volatiles at 30 °C–110 °C, (2) endothermic decomposition of organic matter at 120–600 °C, and (3) formation of residual ash at 600–800 °C in the raw plant material and the *E. hyemale* extract (Figure 1). Figure 1A shows the heat flux measured as a function of increasing temperature applied to the *E. hyemale* extract. The raw plant material contains a greater amount of water (8.2 ± 0.4%) and organic matter (65.3 ± 4.5%) than the extract (3.3 ± 1.0% and 47 ± 2.7%, respectively). A slow decrease in weight may indicate the presence of several volatile components in the region. In contrast, a rapid decrease in weight usually indicates the abundance of a particular component [23]. 

In general, plant extracts are a mixture of substances that interact with each other. In this sense, the decomposition of the extract can be associated with a wide variety of secondary metabolites, mainly phenolic, present in the extract [24]. The extract has a higher thermal stability than the raw plant material because the plant has fibers and increased hydrophilic components that can absorb moisture [25]. In addition, the *E. hyemale* extract contains a slightly higher residual ash mass (consisting of the elements analyzed by X-ray fluorescence) than the raw plant material. These differences are probably due to the extraction process. 

The first derivative analysis of the TGA thermogram shows that the raw material experiences a greater mass loss between 200 and 400 °C (Figure 1B). The mass loss suffered by the samples can be interpreted as a loss of residual solvents, loss of hydration, or decomposition of organic matter [26]. There is also a higher formation of residual ashes between 600 and 800 °C, which is related to the high content of components and inorganic ions of the extracellular matrix of the plant. It is interesting to note that the extraction process carried out allows for obtaining low-molecular-weight organic molecules that are decomposed between 150 and 200 °C, indicating that the ultrasound-/microwave-assisted extraction releases these agents from the plant matrix, obtaining a significant removal of the phytochemical components of the plant extracellular matrix. 

### 2.3. Evaluation of Surface Crystallinity by X-Ray Diffraction 

**Figure 2:**
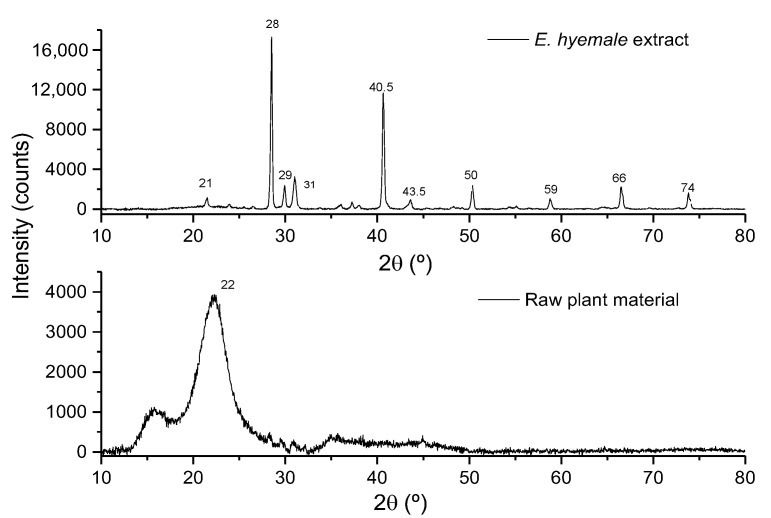
X-ray diffractogram of the extract and raw plant material of *E. hyemale*.

The X-ray diffraction (XRD) technique provides information on the structural state of powder samples, allowing the identification of crystalline, amorphous, and mixed phases. In the diffractogram of *E. hyemale* extract (Figure 2), X-ray diffraction peaks of a higher intensity were observed at angles 2θ = 28.1° and 40.5°, and peaks of a lower intensity were observed at angles of 21.2°, 29.6°, 30.6°, 43.5°, 50°, 59°, 66°, and 74°, indicating that it has a crystalline structure. This can be related to the molecular diffusion phenomena that the extraction process regulates, in addition to the fact that the extracted molecular components can be related to each other, generating surfaces of a crystalline nature. On the other hand, in the diffractogram of the raw plant material, an amorphous halo centered at 22° was observed, indicating that the presence of fibers reduces the surface crystallinity. The presence of biochemical and inorganic components allows the generation of surfaces of an amorphous nature in the raw material. Profiles similar to ours have been reported in other plants where it is mentioned that the peak around 15° corresponds to an amorphous region. The peak around 22° is attributed to crystalline regions related to the cellulose polymer, the main component of plant walls [25,27]. The crystalline structure of *E. hyemale* extract suggests that formulations prepared with the extract powder will be stable and have high bioavailability. Future experiments need to be conducted to address these issues.

### 2.4. Chemical Structure of E. hyemale Extract Evaluated by UV/Vis Spectrophotometry and Fourier Transform Infrared Spectroscopy (FTIR) 

**Figure 3:**
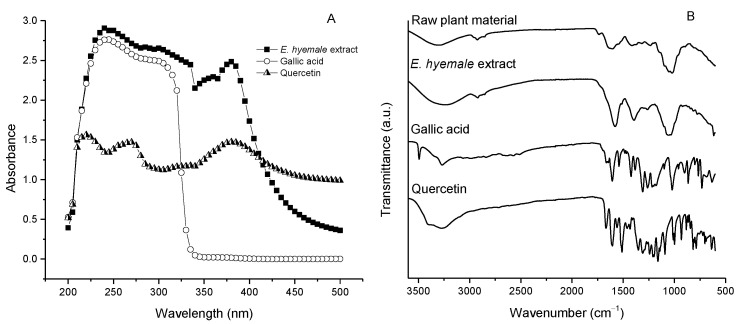
(**A**) UV/Vis absorption spectrum of *E. hyemale* extract, gallic acid, and quercetin. (**B**) FTIR spectrum of raw plant material of *E. hyemale* extract, quercetin, and gallic acid.

The UV/Vis absorption spectrum of *E. hyemale* extract shows two absorption bands between 230 and 270 nm and 340 and 380 nm and a maximum near 400 nm (Figure 3A). The spectrum of gallic acid shows two absorption bands between 240 and 280 nm and 280 and 320 nm, while the spectrum of quercetin shows three absorption bands between 240 and 250 nm, 250 and 280 nm, and 340 and 410 nm (Figure 3A). Typically, the UV/Vis absorption spectrum of flavonoids and phenolic acids is defined by two absorption bands between 300 and 380 nm and 240 and 280 nm intervals [28]. In this sense, the presence of both types of compounds is confirmed by these two characteristic bands in the UV/Vis spectrum of the extract (Figure 3A). These absorption bands are associated with the movement of electrons from π orbitals (aromatic forms) to different molecular orbitals of characteristic energy. 

The FTIR (Fourier transform infrared spectroscopy) spectra (Figure 3B) of the raw plant material and the *E. hyemale* extract are similar, and of these, the broad band between 3400 cm^−1^ and 3200 cm^−1^ corresponds to an O–H bond stretching vibration, indicating the presence of hydroxyl groups. These groups are attributed to the hydroxylated molecules (such as polyphenols) and their water content [6]. The presence of a band of lower intensity in the region between 2935 and 2915 cm^−1^ represents an N–H bond stretching vibration, indicating the presence of molecules with amino groups present in the plant extracellular matrix, as well as those corresponding to the C–H bond vibrations of the CH_2_ group, typical of organic matter. The bands in the region between 1600 and 1475 cm^−1^ indicate the presence of C=C bonds, which are abundant in aromatic compounds with a phenyl nucleus. In the region between 1420 cm^−1^ and 1200 cm^−1^, the band for the C–H bond bending vibration of methyl groups (–CH_3_) and carboxylic acids is found. 

Furthermore, the peaks between 1200 cm^−1^ and 1025 cm^−1^ are probably related to the presence of C–N amine groups, as observed in Figure 3B. The bands between 1000 and 900 cm^−1^ are related to the C-O-C bond vibrations of possible carbohydrates remaining in the extract. These results suggest that the extraction process does not alter the functional group content of the extract with respect to the raw plant material of *E. hyemale*. However, the intensity of the bands in the extract is higher than in the raw plant material, indicating a higher presence of bioavailable phytochemical compounds in the extract [29]. In addition, the release of these phytochemical components from the extracellular matrix of the plant by the extraction process promotes that these components have a higher vibration in the infrared than when they are trapped in the plant. 

FTIR spectra of the gallic acid and quercetin standards were also obtained. Some similarities were observed between the spectra of the extract and these standards, especially in the regions consisting of OH groups, aromatic groups, carboxylic groups, and ether groups. However, other bands (800–1500 cm^−1^) were also observed in quercetin and gallic acid that were not observed in the extract, proving that they are components of lower polarity (hydrophobic) that are not extracted by the process used. 

### 2.5. Total Polyphenol and Flavonoid Content in E. hyemale Extract 

The content of total phenolic compounds in *E. hyemale* extract is 41.9 ± 2.4 mgEAG/g (mg equivalents of gallic acid per gram of sample), while the content of flavonoids is 5.5 ± 2.7 mgEQ/g (mg equivalents of quercetin per gram of sample). The phenolic compounds of *E. arvense* extract [30] are four times higher than those of *E. hyemale*. On the other hand, the concentration of flavonoids is similar to that found in *E. arvense* in Brazilian regions [7]. A variation in the concentrations of total polyphenols in *E. hyemale* could be due to the differences in the extraction procedures, the plant material used, as well as the different geographical locations of the *Equisetum* plants [7,31]. The gallic-acid-like phenolic and quercetin-like flavonoid compounds in *E. hyemale* suggest that the extract may have anti-inflammatory and antimicrobial activities similar to other *Equisetum* plants from different regions of the world.

### 2.6. Identification of Compounds Contained in E. hyemale Extract by Chromatography 

Gas chromatographic (CG-MS) analysis revealed the presence of some components derived from sterols in the extract of *E. hyemale* (Table 2), such as campesterol, cycloartenol, γ-sitosterol, and a phenylpropenoid such as phenol-2,4-bis(1-phenylethyl). γ-sitosterol [32,33] and phenol-2,4-bis(1-phenylethyl) [34,35] these components have anti-inflammatory properties. Cycloartenol has anti-inflammatory effects by inhibiting cell proliferation. In addition, it also shows antioxidant and antitumor responses [36,37]. Campesterol reduces total cholesterol and low-density lipoprotein (LDL) blood levels [38]. 

On the other hand, reverse-phase high-performance liquid chromatography (RP-HPLC-ESI-MS) analysis revealed the presence of three flavonols (kaempferol 3,7,4’-O-triglucoside, kaempferol 3,7-O-diglucoside, and quercetin), two phenolic acids (caffeic acid 4-O-glucoside and ferulic acid 4-O-glucoside), and one lignan (conidendrin) in *E. hyemale* extract (Table 3). Similar phytochemicals have been identified in *E. hyemale* fractions obtained with ethanol, methanol, diethyl ether, n-hexane, dichloromethane, and ethyl acetate using percolation, maceration, and ultrasound, in addition to caffeoyl tartaric acid, chlorogenic acid, cinnamic acid, luteolin, various flavones, unsaturated fatty acids, and carotenoids [5,7,13,39]. It is noteworthy that, with our extraction procedure (ultrasound and microwave), we identified phenol-2,4-bis(1-phenylethyl) and conidendrin, phenolic compounds that have never been reported in *E. hyemale* or other plants of the genus *Equisetum*. The presence of hydroxyl groups in the identified molecules allows their successful extraction, but not those with low polarity. The phenolic compounds identified in Mexican *E. hyemale* have pharmacological properties, including antimicrobial, antioxidant, anti-inflammatory, antiviral, and antimutagenic activity, among others.

### 2.7. Effect of E. hyemale Extract on the Viability of RAW 264.7 Cells and Porcine Skin Fibroblasts 

**Figure 4:**
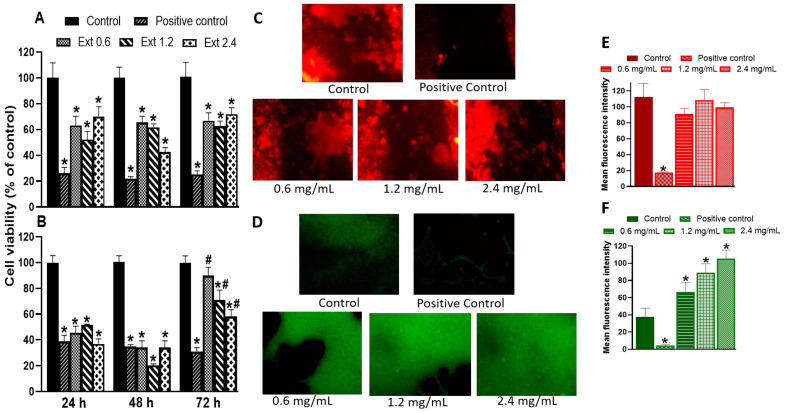
Effect of *E. hyemale* extract (Ext: 0.6, 1.2, and 2.4 mg/mL) on the viability of RAW 264.7 cells (**A**) and porcine skin fibroblasts (**B**) after 24, 48, and 72 h of treatment. (**C**,**D**) Representative micrographs of the proliferation assay of RAW 264.7 cells and skin fibroblasts stained with rhodamine B and fluorescein, respectively. (**E**,**F**) Quantification of fluorescence signal in RAW 264.7 cells and fibroblasts, respectively. Positive control corresponds to the cell treatment with triton X-100 (1%). * *p* < 0.05 versus control (DMEM), # *p* < 0.05 versus the same concentration at 24 and 48 h.

As expected, triton treatment (positive control) reduced the viability of the murine macrophage cell line, RAW 264.7 cells (Figure 4A), and porcine skin fibroblasts by 80 and 60%, respectively (Figure 4B). In RAW 264.7 cells, the *E. hyemale* extract reduced the cell viability by 30% to 60% at the three concentrations tested (0.6, 1.2, and 2.4 mg/mL) and at all times evaluated, but this effect was not dependent on the concentration or incubation time. On the other hand, the viability of porcine skin fibroblasts was significantly reduced (60–80%) at 24 and 48 h with all concentrations tested. However, after 72 h of treatment with the extract, fibroblast viability remained unchanged at the lowest concentration (0.6 mg/mL). At higher concentrations (1.2 and 2.4 mg/mL), the viability was reduced by only 30–40% compared with the control, suggesting that cytotoxicity on fibroblasts may depend on the incubation time. That is, low cytotoxicity can be observed with longer extract incubation times with the extract. Consistent with this, it has been reported that *E. hyemale* ethanolic extract has low cytotoxicity and maintains approximately 75% Vero cell viability at concentrations up to 12.5 mg/mL [40]. It has also been reported that *E. hyemale* extract inhibits L1210 cell proliferation by inducing G2/M arrest and cell apoptosis [41]. In addition, the ethyl acetate extract has a low toxicity in mice, causing mild changes in the liver without causing necrosis [5]. 

It should be noted that 3-(4,5-dimethyl-2-thiazolyl)-2,5-diphenyl-2H-tetrazolium bromide (MTT) is reduced to formazan salts in mitochondria. This reduction depends on several factors: concentration, incubation time, number of viable cells, and mitochondrial metabolic activity. In this sense, the reduced viability found in RAW 264.7 cells and fibroblasts could also be due to the fact that adherent cells proliferate and growth is inhibited by contact so that mitochondrial metabolism (viability) can be reduced [42]. Our proliferation data support this idea, at least for fibroblasts. As shown in Figure 4C,E, the extract did not alter the proliferation rate of RAW 264.7 cells, indicated by red fluorescence. However, the extract increased the green fluorescence in skin fibroblasts, indicating a higher proliferation rate of this cell type (Figure 4D,F). In both cases, triton treatment reduced the proliferation of both cell types, suggesting that skin fibroblasts are more sensitive than macrophages to some compounds of the extract that modulate biological targets involved in the viability or proliferation phenomena [43]. At this time, we do not identify the specific compound of the extract that mediates these effects on macrophage and fibroblast viability and proliferation. However, it has been shown that γ-sitosterol inhibits cell growth and cell cycle arrest of MCF-7 and A549 cells [44], phenol-2,4-bis(1-phenylethyl) inhibits human neutrophil migration [35], caffeic acid inhibits fibrosarcoma cell proliferation [45], kaempferol reduces fibroblast proliferation [46], and ferulic acid inhibits proliferation of RAW 264.7 cells [47]. In contrast, quercetin enhances fibroblast proliferation [48]. Cycloartenol and conidendrin have cytoprotective effects against cytotoxic stimuli in pancreatic and SH-SY5Y cells [49,50], and campesterol does not affect the RAW 264.7 cell viability [51]. Future work is needed to analyze the role of each component or their combinations.

### 2.8. Effect of E. hyemale Extract on the Release of Inflammatory Mediators from RAW 264.7 Cells 

**Figure 5:**
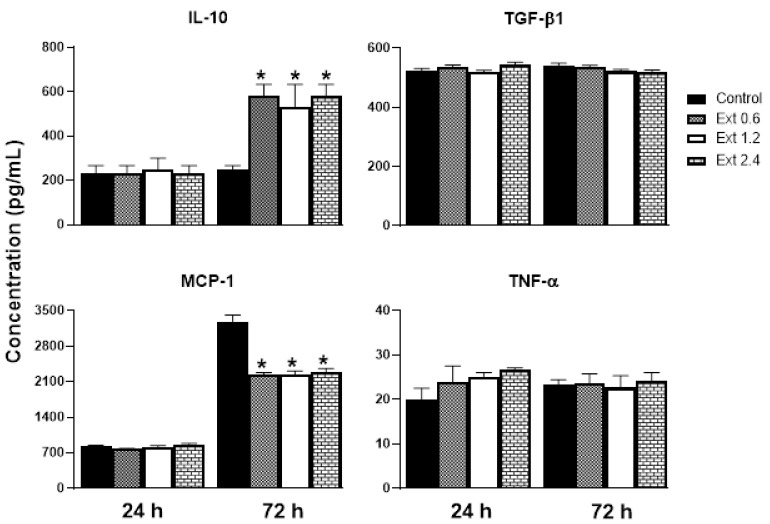
Effect of *E. hyemale* extract (Ext: 0.6, 1.2, and 2.4 mg/mL) on IL-10, TGF-β1, MCP-1, and TNF-α release from RAW 264.7 cells stimulated with LPS (*lipopolysaccharide*) after 24 and 72 h of treatment. * *p* < 0.05 versus control.

Figure 5 shows the release of inflammatory mediators from RAW 264.7 cells stimulated with lipopolysaccharide (LPS). In this sense, treatment with *E. hyemale* extract did not affect the release of transforming growth factor-beta1 (TGF-β1) and TNF-α from RAW 264.7 cells at the times evaluated. However, the release of interleukin-10 (IL-10) was increased (threefold) only up to 72 h after stimulation with the extract, while the release of monocyte chemoattractant protein-1 (MCP-1) was decreased. It should be noted that the release of MCP-1 from control cells was fivefold higher at 72 h than at 24 h. This cytokine release pattern induced by the extract is not dependent on its concentration.

IL-10 is a potent anti-inflammatory cytokine that facilitates wound healing by improving the transition from the inflammatory phase to the proliferative phase in injured tissue. It has also been documented that IL-10 reduces MCP-1 levels [52]. Under our experimental conditions, it is likely that MCP-1 levels were reduced by the increased release of IL-10. MCP-1 is a protein that attracts monocytes from the blood and macrophages from the tissue, releasing proinflammatory cytokines, thereby establishing a chronic inflammation and delaying the healing process. Therefore, it is convenient that *E. hyemale* extract inhibits the release of MCP-1. However, some evidence suggests that MCP-1 promotes wound epithelialization (proliferation phase) [52], so its release needs to be reduced but not completely inhibited. It is important to note that, although MCP-1 release from RAW 264.7 cells is reduced, its release is two times higher than after 24 h. These effects of *E. hyemale* extract on MCP-1 release may balance its low cytotoxic effect on macrophages and its proinflammatory effect. These results suggest that *E. hyemale* extract may modulate the release of IL-10 and MCP-1 to facilitate faster wound healing. At this time, we do not know the specific compound responsible for these inflammatory mediator release profiles. However, we believe that cycloartenol, γ-sitosterol, phenol-2,4-bis(phenylethyl), flavonoids, and polyphenols contained in the *E. hyemale* extract may be involved. Flavonoids such as quercetin and kaempferol promote the secretion of anti-inflammatory cytokines and inhibit the release of inflammatory secretions through up- and downregulation of multiple pathways [53]. The role of campesterol in this effect seems unlikely as it does not alter IL-10 release from macrophages [54] or T lymphocytes [55]. 

On the other hand, TGF-β1 is a cytokine that induces the proliferation of fibroblasts and extracellular matrix proteins, favoring wound healing, and TNF-α is a potent proinflammatory cytokine that promotes the establishment of chronic inflammation [56]. In this sense, *E. hyemale* extract did not modify the TGF-β1 and TNF-α release in RAW 264.7 cells. Regarding TNF-α, low concentrations (below 30 pg/mL) were quantified in this work compared with other studies [57,58]. Only one study has reported similar concentrations [59]. These differences in the magnitude of the TNF-α concentration release could be due to, among other factors, incubation time, culture medium, cell line passage, treatment protocols, and regulation by other cytokines in the medium [60,61]. These results suggest that *E. hyemale* extract has anti-inflammatory properties by increasing the IL-10 release and inhibiting MCP-1 release at 72 h, without altering the release of TGF-β1 and TNF-α from RAW 264.7 cells.

The exact events that produce this modulatory pattern of macrophage inflammatory cytokine release are unclear. Again, as mentioned above, this pattern could be due to the combination of the individual effects of the phytochemicals in the extract. Thus, (1) γ-sitosterol reduces cell proliferation [44]; (2) phenol-2,4-bis(1-phenylethyl) is associated with inhibition of superoxide anion production by N-formyl-methionyl-leucyl-phenylalanine (fMLP) [35]; (3) caffeic and ferulic acids, campesterol, and quercetin inhibit nuclear factor kappa B (NF-κB) and mitogen-activated protein kinases (MAPK) such as p38 MAPK and JNK1/2 [47,62,63,64]; and (4) kaempferol reduces inflammatory cytokine expression and cell proliferation [46,65]. 

### 2.9. Antimicrobial Activity of E. hyemale Extract 

**Figure 6:**
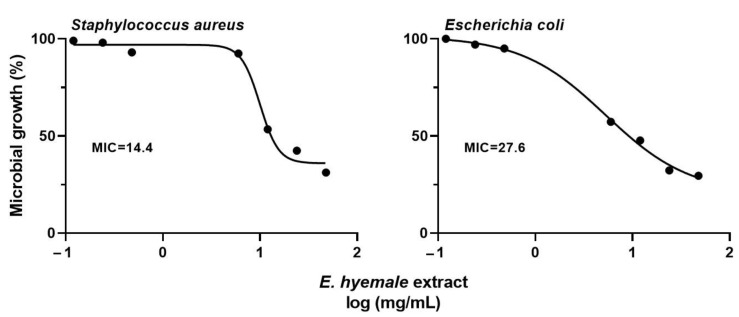
Effect of *E. hyemale* extract on the microbial growth of *Staphylococcus aureus* and *Escherichia coli* after 48 h of treatment. Minimum inhibitory concentration (MIC: mg/mL).

The antimicrobial activity of *E. hyemale* extract was evaluated against *S. aureus* (Gram-positive) and *E. coli* (Gram-negative) (Figure 6). Predictably, ceftriaxone induced a potent antimicrobial effect (minimum inhibitory concentration, MIC = 1 mg/mL) against both bacterial strains, as has been reported [66]. In addition, the *E. hyemale* extract inhibited the growth of *S. aureus* (MIC = 14.4 mg/mL) and *E. coli* (MIC = 27.6 mg/mL), with better potency against *S. aureus* than against *E. coli*.

Our results show that the extract from the Mexican *E. hyemale* has similar antimicrobial potency to the extract from the Brazilian plant (MIC = 13.1 mg/mL for both bacterial strains) [7,9], but better antimicrobial potency than the European *Equisetum* plant (MIC = 100 mg/mL for both strains) [67]. The separation technique, the solvents, and the growth zone of the plant influence the pharmacological efficacy of its bioactive compounds. For example, the extract from the Brazilian plant was obtained by maceration and using 70% v/v of ethanol. In this work, we used an ultrasound-/microwave-assisted extraction with 40% v/v ethanol, while the European extract was obtained with 70% v/v methanol. These differences could influence on the content of phenolic compounds (antioxidants) associated with the antimicrobial activity of *E. hyemale* extract [5,6,7,8], such as quercetin and kaempferol [7,8,9,67,68]. Curiously, the content of flavonoids is similar in both Mexican and Brazilian plants, suggesting that these bioactive compounds are responsible for their antibacterial activities. However, metallic elements such as iron and magnesium in the Mexican *E. hyemale* extract could also be involved in its antibacterial activity [22]. 

Microbial infections delay wound healing and *S. aureus* is the major infecting strain. Therefore, it is relevant that *E. hyemale* extract has antimicrobial activity against this bacterium. However, the antimicrobial effects of *E. hyemale* extract occur at concentrations between 10 and 30 times higher than those with anti-inflammatory activity. Therefore, the next steps in the research of *E. hyemale* extract will focus on improving its antimicrobial potential. This challenge can be addressed by (1) improving the extraction process to concentrate the antimicrobial compounds [8,69], (2) combining the extract with compounds with high antimicrobial potency [70], and (3) incorporating the extract into polymeric matrices that enhance its antimicrobial properties [71]. After achieving the above, new studies are needed to investigate the antimicrobial activity of the extract on clinical isolates of bacterial strains of *S. aureus*, *S. epidermis*, *P. aeruginosa*, *Streptococcus* spp., and *Enterococcus* spp.

### 2.10. Effect of E. hyemale Extract on Wound Healing in Type 2 Diabetic Rats

Blood glucose levels were 103 ± 7 mg/dL in non-diabetic rats and 298 ± 17 mg/dL in diabetic rats on day 0. After 21 days, these levels were 105 ± 13 and 400 ± 18 mg/dL for non-diabetic and diabetic rats, respectively. The above values indicate the establishment and maintenance of type 2 diabetes in the animals studied.

**Figure 7:**
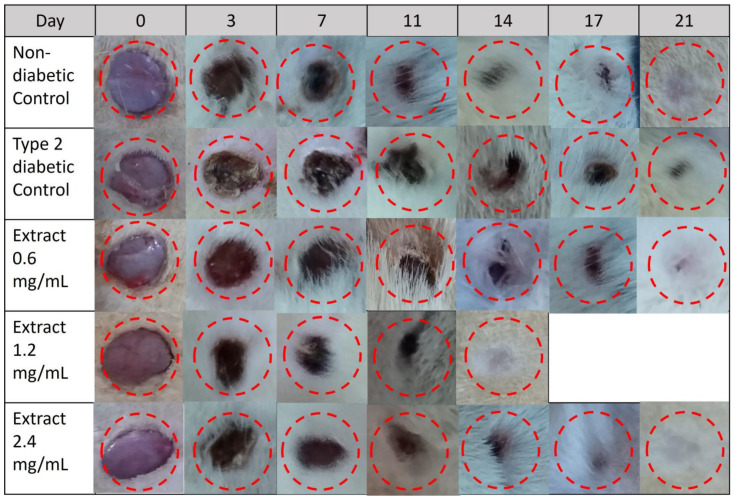
Representative photographs of the effect of *E. hyemale* extract (0.6, 1.2, and 2.4 mg/mL) on wound closure during 21 days in type 2 diabetic rats. Non-diabetic control and type 2 diabetic control groups were treated with physiological saline (0.9%).

Figure 7 shows the time course of the wound-healing progress in Wistar rats. All experimental groups showed progressive wound reduction from day 3 to day 21. Visually, the wounds showed no signs of excessive redness, irritation, or infection. In the non-diabetic control group treated with physiological saline solution (0.9%), the wound healed completely by day 21. However, delaying healing was observed in type 2 diabetic rats receiving the same treatment (wound slightly open on day 21). In fact, in this group, the wound did not close until day 28. On the other hand, daily application of the lowest concentration (0.6 mg/mL) of the extract did not accelerate the wound-healing process in diabetic rats, but the treatment with concentrations of 1.2 mg/mL and 2.4 mg/mL accelerated the healing process, as the wounds were closed completely on days 14 and 21, respectively. 

**Figure 8:**
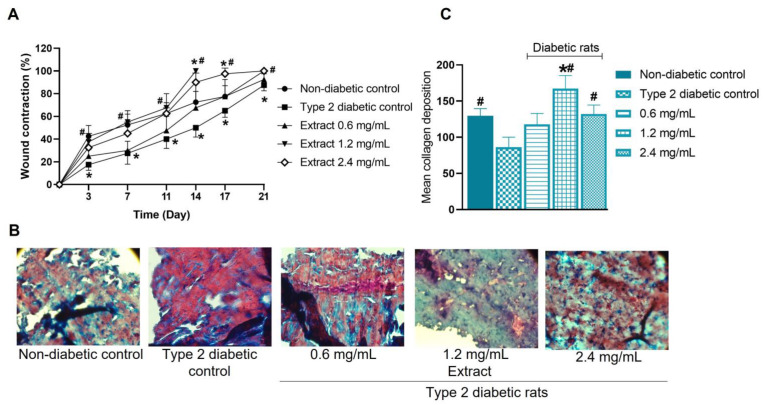
Wound-healing rate (**A**) of type 2 diabetic rats treated with *E. hyemale* extract (0.6, 1.2, and 2.4 mg/mL) for 21 days. Non-diabetic and type 2 diabetic control groups were treated with physiological saline (0.9%). (**B**) Representative images of healed skin from type 2 diabetic rats were obtained on days 21, 28, 24, 14, and 21 for the non-diabetic group, diabetic group, and diabetic group treated with 0.6, 1.2, and 2.4 mg/mL of the extract, respectively, and stained with Masson’s trichrome. The image shows collagen structures formed in the dermal layer (blue: collagen; red: cytoplasm; dark/blue: cell nuclei). The magnification corresponds to ×40. (**C**) Quantification of collagen deposition. * *p* < 0.05 versus non-diabetic control group; # *p* < 0.05 versus type 2 diabetic control group.

The graph of wound healing as a function of time is shown in Figure 8A (wound contraction). On days 3, 7, 11, and 14, the healing process in diabetic rats was slower than in non-diabetic rats (treated with physiological saline solution). In fact, the percentage of wound contraction in diabetic rats on these days was approximately 50% less than in non-diabetic rats. On days 17 and 21, the rate of wound contraction in diabetic rats was still lower than in non-diabetic rats, although the contraction difference was only 13%. 

Application of the extract at 0.6 mg/mL showed a tendency to accelerate the healing process in diabetic rats over time, although there was not statistically significant difference. Concentrations of 1.2 and 2.4 mg/mL of the extract significantly improved wound healing in type 2 diabetic rats, as wound contraction (30–40%) occurred similarly in non-diabetic rats on days 3, 7, and 11, but, from day 14 to 17, the wound contraction was better in diabetic rats treated with higher concentrations of the extract than in saline-treated non-diabetic and diabetic rats. The 1.2 mg/mL concentration of *E. hyemale* extract induced complete wound healing at 14 days, while the 2.4 mg/mL concentration did so up to 21 days. Although the highest concentration used induced faster wound healing than in diabetic rats treated with saline alone, we expected a reduction in the healing process less than 14 days, which did not occur. The simplest way to explain this phenomenon is that, as is the case with complex mixtures [72], the extract could exhibit hormetic responses (inverted U), whereby, at intermediate concentrations, the ratio of each component favors faster healing through a synergy between their phytochemicals. 

After complete wound healing, the skin was harvested on days 21, 28, 24, 14, and 21 for the non-diabetic, diabetic control, and diabetic groups treated with 0.6, 1.2, and 2.4 mg/mL of the extract, respectively. Masson’s trichrome staining revealed that the skin of non-diabetic and diabetic rats showed cell infiltrates and thin collagen bundles with a regular arrangement (Figure 8B). However, in the skin of type 2 diabetic rats, the cell infiltrate was higher than in the skin of non-diabetic rats. In contrast, the collagen content was reduced (Figure 8C), suggesting less migration of fibroblasts into the injured skin. Considering this, we believe that the cell infiltrates formed by a higher proportion of inflammatory cells, such as macrophages, lead to delayed healing in this group. On the other hand, the dermal layer of the healed skin of type 2 diabetic rats treated with the extract was similar to that of non-diabetic rats. However, some differences were observed. Regarding collagen deposition, the treatment with concentrations of 0.6 and 2.4 mg/mL (Figure 8C) did not modify this parameter. Although the treatment with 1.2 mg/mL significantly increased the collagen content in the healed skin, treatment with 2.4 mg/mL of extract produced the most compact collagen layer. Cell infiltration in the skin of rats treated with 0.6, 1.2, and 2.4 mg/mL of the extract appeared to be higher than that of non-diabetic rats, but lower than that of diabetic rats treated with physiological saline alone. These results suggest that treatment with *E. hyemale* extract accelerates skin healing in type 2 diabetic rats by promoting the migration of fibroblasts into the damaged tissue. The main limitation of the present work is that histological analysis was performed on the skin of type 2 diabetic rats treated with the extract until the wound was completely healed. Future work must be performed at different times after wounding to know specific changes in the healing phenomenon induced by the extract of *E. hyemale*. 

Some flavonoids and γ-sitosterol have been shown to have potential as wound-healing agents [73,74,75,76,77]. The wound-healing properties of quercetin, caffeic and ferulic acids, and campesterol are related to the modulation of inflammatory cytokines and enhancement of fibroblast proliferation [78,79,80] via the Wnt/β-catenin signaling pathway [48,78]. In addition, cycloartenol [81] and γ-sitosterol promote collagen synthesis and fibroblast migration into injured tissue [76,77], whereas kaempferol delays healing by inhibiting the TGF-β1 receptor [46]. On this basis, it is likely that quercetin, γ-sitosterol, campesterol, cycloartenol, caffeic acid 4-O-glucoside, and ferulic acid 4-O-glucoside of the extract are involved in the wound-healing effect of *E. hyemale* extract.

Finally, from a simple perspective, a potential wound-healing treatment must reduce the viability/function of macrophages (to modulate the inflammatory phase of wound healing) and increase the proliferation of the fibroblasts (to accelerate the proliferative and remodeling phases) in the injured tissue. The *E. hyemale* extract meets these requirements. However, some negative reactions may occur with the topical application of the extract, so it is recommended not to use the horsetail extract in the case of suspected hypersensitivity to some of the active ingredients such as phenolic acids, flavonols, and sterols.

## 3. Materials and Methods

### 3.1. Chemical Reagents and Materials 

The compounds used in this study were gallic acid (3,4,5-trihydroxybenzoic acid), quercetin (3,3′,4′,5,6-pentahydroxyflavone), Folin–Ciocalteu reagent, 3-(4,5-dimethyl-2-thiazolyl)-2,5-diphenyl-2H-tetrazolium bromide (MTT), ceftriaxone disodium salt hemi (heptahydrate), barium chloride dihydrate, sodium carbonate, aluminum chloride, sodium acetate, acetic acid, methanol, sulfuric acid, lipopolysaccharides (LPS) from *E. coli* O111:B4, rhodamine, fluorescein, and interleukin-10 (RAB0245-1KT, IL-10). Tumor necrosis factor-a (RAB0477-1KT, TNF-a) ELISA kits were purchased from Sigma Chemical Co., St. Louis, MO, USA. Transforming growth factor-b1 (BMS608-4, TGF-b1) and monocyte chemoattractant protein-1 (BMS6005, MCP-1) ELISA kits were purchased from Invitrogen, Walthman, MA, USA. Streptozotocin (STZ) was purchased from Cayman Chemical, Ann Arbor, MI, USA. 2-Propanol was purchased from J.T. Baker, CDMX, Mexico. Pentobarbital was purchased from Aranda-Salud Animal, Guadalajara, JAL, Mexico. Trichrome staining kit was purchased from Hycel, Guadalajara, JAL, Mexico. Triton X-100 and nicotinamide were purchased from Bio Basic Inc., Markham, ON, Canada. Dulbecco’s modified eagle medium (DMEM), trypsin 1X, fetal bovine serum, and penicillin-streptomycin solution 50X were purchased from Corning, Glendale, AZ, USA. RAW 264.7 cells (ATCC TIB-71, lot 70026471) were purchased from American Type Culture Collection (ATCC, USA). *S. aureus* (ATCC 23235) and *E. coli* (ATCC 25922) bacterial strains were donated by the Microbiology Laboratory of the Chemistry Department of the Autonomous University of Coahuila. 

### 3.2. Plant Material 

*Equisetum hyemale* var. *affine* (Engel) A.A Eaton plant (horsetail, carricillo) was collected in July 2020, within the borders of the states of Jalisco and Zacatecas, Mexico (21°15′12.2″ N 102°52′07.1″ W). Certified botanists identified the plant and a specimen was deposited in the Luz María Villarreal de Puga Herbarium (voucher SIS-TRA-2020-03). The stem of the plant was dried in an oven at a temperature of 45 °C for 48 h and pulverized until a fine powder with a homogeneous particle size was obtained. 

### 3.3. Elemental Analysis of Raw Plant Material by X-ray Fluorescence (XRF) 

Elemental analysis of the raw plant material was performed by the X-ray fluorescence (XRF) method in a Malvern-PANalytical X-ray spectrometer at a voltage of 40 kV and a current of 30 mA, with a CuKα X-ray source (λ = 1.54 Å). 

### 3.4. Extraction Procedure 

For this, we used the powder obtained from the plant raw material in an ethanolic solution in a ratio of 1:10 (powder–solvent). Briefly, the extraction was performed with 80 g of powder and 800 mL of 40% v/v ethanol in a 1 L reactor. The reactor was connected to an ultrasound and microwave cooperative workstation (ATPIO Instruments Manufacture Co., Ltd., Nanjing, China). The instrument was operated in ultrasound mode at an amplitude of 25 KHz with a power of 99% at 28 °C. In microwave mode, the device was operated at 800 W and 50 °C. The equipment was operated simultaneously for 30 min. The extract was filtered and then dried at room temperature. The extract was stored frozen (−20 °C) until use. Three different lots were used for phytochemical screening and biological activities. 

### 3.5. Thermogravimetric Analysis (TGA) of Raw Material and Extract 

The analysis of the raw material of the plant and the extract was carried out using a thermogravimetric analyzer (TGA-4000, Perkin Elmer, Shelton, CT, USA). The operating conditions used were a temperature range of 30 to 800 °C, a heating rate of 20 °C/min, and the use of nitrogen as an inert atmosphere up to 600 °C (20 mL/min); subsequently, a change from a gaseous atmosphere to oxygen was made to accelerate the exothermic decomposition of the analyzed samples up to 800 °C (20 mL/min). 

### 3.6. X-ray Diffraction (XRD) Evaluation of the Raw Material and Extract 

X-ray scattering measurements were performed on the extract and raw plant material of *E. hyemale* using Malvern-PANalytical equipment (Malvern, Worcs, UK) at a voltage of 40 kV and a current of 30 mA, with a CuKα X-ray source (λ = 1.54 Å) in the 2θ range from 10 to 80°. 

### 3.7. UV/Vis Spectrophotometry and Fourier Transform Infrared Spectroscopy (FTIR) Analysis of the Extract

The extract of *E. hyemale* (10 mg/mL) dissolved in water was scanned with a spectrophotometer (Metash Instruments Co. Ltd, Shangai, China) at a wavelength of 200 to 700 nm. Gallic acid and quercetin standards (10 mg/mL) were used for comparison. On the other hand, infrared analysis of raw plant material, *E. hyemale* extract, and gallic acid and quercetin standards was performed. Frontier equipment (Perkin Elmer, Shelton, CT, USA) was used, and the spectra were recorded with an attenuated total reflectance (ATR) accessory, with a resolution of 16 cm^−1^ in the range of 4000 to 650 cm^−1^, using an average of 16 scans. 

### 3.8. Quantification of Total Polyphenols and Flavonoids in the Extract 

Quantification of total polyphenols in the extract was performed according to the modified Folin–Ciocalteu method. For this, 10% v/v Folin–Ciocalteu reagent, 2% w/v Na_2_CO_3_ solution, and a 10 mg/mL solution of the extract in water were prepared. A 96-well microplate was filled with 40 µL of the extract, 80 µL of 2% w/v sodium carbonate, and 100 µL of the Folin–Ciocalteu reagent. The mixture was incubated for 15 min at room temperature. The absorbance of the sample was measured at 765 nm in a Sinergy HTX spectrophotometer (Fisher Scientific, Waltham, MA, USA). A modified aluminum chloride colorimetric method was used to determine the flavonoid content. For this purpose, 0.1 mL of a solution of 10 mg/mL of the extract in water, 1.4 mL of water, and 0.50 mL of the flavonoid reagent were mixed. The flavonoid reagent was prepared with 133 mg of aluminum chloride and 400 mg of sodium acetate in 100 mL of the solvent (140 mL of methanol, 50 mL of water, and 10 mL of acetic acid). Once the solutions were prepared, a volume of 200 µL of each sample or standard were transferred to a 96-well microplate and kept at room temperature for 30 min. Absorbance was measured at 430 nm in a Sinergy HTX spectrophotometer (Fisher Scientific, Waltham, MA, USA). A standard curve was constructed for gallic acid (7.8, 15.6, 31.3, 62.5, 125, 250, and 500 μg/mL) and quercetin (7.8, 15.6, 31.3, 62.5, 125, 250, 500, and 1000 μg/mL). Total polyphenols and flavonoids were expressed as mg gallic acid or quercetin equivalents per gram of sample (mgE/g), respectively. Three different lots were analyzed and three replicates of each lot were performed. 

### 3.9. Identification of Components of the Extract by Chromatography

#### 3.9.1. Gas Chromatography (CG-MS)

Chromatographic analysis was carried out on a Varian^®^ Gas chromatography system (CP3800; Varian Inc., Walnut Creek, CA, USA) coupled with a Saturn 200 mass spectrometer (Varian Inc., Walnut Creek, CA, USA). The HP-5 MS column with a film thickness of 0.25 μm (30 m × 0.25 mm) was used for separation. The carrier gas was helium at a flow rate of 1 mL/min. The temperature of the injector and detector was 250 °C. MS detector parameters were transferred at a line temperature of 290 °C, with a solvent lag of 3 min. MS was performed in scan mode (m/z 40–600) for qualitative analysis. The identities of the components of the extract were assigned by the comparison of their retention times and mass spectra fragmentation patterns with those stored in the computer kit library, the NIST mass spectra library, and the published literature. Three different lots were analyzed. 

#### 3.9.2. Reverse-Phase High-Performance Liquid Chromatography (RP-HPLC-ESI-MS)

A Varian HPLC system including an autosampler (Varian ProStar 410, Walnut Creek, CA, USA), a ternary pump (Varian ProStar 230I, Walnut Creek, CA, USA), and a PDA detector (Varian ProStar 330, Walnut Creek, CA, USA) were used for reverse-phase high-performance liquid chromatography analysis, as previously reported [82]. Briefly, a liquid chromatograph ion trap mass spectrometer (Varian 500-MS IT Mass Spectrometer, Walnut Creek, CA, USA) equipped with an electrospray ion source was also used. Samples (5 µL) were injected onto a Denali C18 column (150 mm × 2.1 mm, 3µm, Grace, ND, USA). The oven temperature was maintained at 30 °C. The eluents were formic acid (0.2% v/v; solvent A) and acetonitrile (solvent B). The following gradient was applied: initial, 3% v/v B; 0–5 min, 9% v/v B linear; 5–15 min, 16% v/v B linear; 15–45 min, 50% v/v B linear. The column was then washed and reconditioned. The flow rate was maintained at 0.2 mL/min and elution was monitored at 245, 280, 320, and 550 nm. The whole effluent (0.2 mL/min) was injected into the source of the mass spectrometer, without splitting. All MS experiments were carried out in the negative mode [M-H]^−1^. Nitrogen was used as the nebulizing gas and helium as the damping gas. The ion source parameters were as follows: spray voltage 5.0 kV and capillary voltage and temperature of 90.0 V and 350 °C, respectively. Data were collected and processed using MS Workstation software (v 6.9). Samples were firstly analyzed in full scan mode acquired in the m/z range 50–2000.

### 3.10. Cell Assays 

#### 3.10.1. Cells

RAW 264.7 cells (ATCC TIB-71) and porcine skin fibroblasts were cultured in Dulbecco’s modified Eagle’s medium (DMEM) supplemented with 10% v/v fetal bovine serum (FBS) and 1% v/v antibiotic (penicillin–streptomycin) and incubated at 37 °C in 5% CO_2_. Three replicates of three independent cultures were performed for cell culture experiments.

#### 3.10.2. Effect of Horsetail Extract on the Viability of RAW 264.7 Cells and Porcine Skin Fibroblasts

RAW 264.7 cells and porcine skin fibroblasts were seeded at 2 × 10^4^ and 5 × 10^4^ cells, respectively, in 96-well plates. The MTT assay was performed according to the manufacturer’s recommendations. Briefly, the cells were incubated with 100 µL of the extract (0.6, 1.2, and 2.4 mg/mL) and controls (triton X-100 at 1% v/v and DMEM medium) for 24, 48, and 72 h at 37 °C and 5% CO_2_. Thereafter, 10 µL of MTT (1% w/v) was added to each well and the plates were incubated for 2 h. Then, 100 µL of 2-propanol was added and the absorbance (abs) was read at 560 nm in a spectrophotometer (Sinergy HTX, Fisher Scientific, Waltham, MA, USA). The percentage of viability was calculated according to Equation (1):

(1) $$\%\;\mathrm{viability}=\frac{\mathrm{abs}\;\mathrm{sample}-\mathrm{abs}\;\mathrm{blank}\;}{\mathrm{abs}\;\mathrm{control}\;\left(\mathrm{DMEM}\right)-\mathrm{abs}\;\mathrm{blank}}\times 100$$

#### 3.10.3. Effect of Horsetail Extract on the Proliferation of RAW 264.7 Cells and Porcine Skin Fibroblasts

Cell proliferation was determined in RAW 264.7 cells and porcine skin fibroblasts stained with rhodamine B and fluorescein, respectively. Briefly, 1 mL of the extract (0.6, 1.2, and 2.4 mg/mL) and 1 mL of RAW 264.7 or fibroblast cell suspension (2 × 10^5^ cells/mL) were mixed and incubated at 37 °C and 5% CO_2_ for 48 h. DMEM was used as the control and Triton X-100 (1% v/v) as a positive control. The cells were then incubated with fluorescent dyes for two hours and transferred to a microscope slide. The fluorescence signal was observed with a fluorescence microscope (VELAB VE-146YT, Pharr, TX, USA). The samples were excited with a green laser for rhodamine (λ = 532 nm) and fluorescein (λ = 427 nm). Ten fields were randomly recorded for each slide and the assays were performed in triplicate. Fluorescence intensity was quantified using freely available ImageJ software (https://imagej.nih.gov/ij/download.html accessed on 3 February 2023). 

### 3.11. Effect of Extract on Cytokine Release from RAW 264.7 Cells 

RAW 264.7 cells were seeded at 5 × 10^4^ cells in Eppendorf tubes. Cells were stimulated with bacterial lipopolysaccharides (LPS, 1 µg/mL) followed by the *E. hyemale* extract at 0.6, 1.2, and 2.4 mg/mL or control. Cell incubation was performed at 37 °C in 5% CO_2_ for 24 and 72 h. At the end of the incubation, the supernatants were collected and frozen at −80 °C until use. Quantification of interleukin-10 (IL-10), tumor necrosis factor-alpha (TNF-α), transforming growth factor-beta1 (TGF-β1), and monocyte chemoattractant protein-1 (MCP-1) was performed by immunoassay following the manufacturer’s instructions. 

### 3.12. Evaluation of Antimicrobial Activity of E. hyemale Extract

The antimicrobial activity of *E. hyemale* extract was evaluated by the broth dilution method. Briefly, a 0.5 McFarland standard was prepared with 0.5 mL of 1.175 w/v barium chloride dihydrate solution and 9.5 mL of a 1% v/v sulfuric acid solution (0.18 mol/L, 0.36 N). The optical density of the turbidity standard was then measured using a spectrophotometer (Metash Instruments Co. Ltd, Shangai, China) with a 1 cm cuvette at a wavelength of 600 nm, and bacterial suspensions of 1.5 × 10^8^ CFU/mL were prepared. An aliquot of the suspension of *S. aureus* and *E. coli* strains was transferred to screw-capped glass tubes containing Müller–Hinton culture broth plus 1 mL of *E. hyemale* extract (0.12–48 mg/mL), resulting in a final concentration of 3 × 10^6^ CFU/mL. The mixture was then incubated at 37 °C with continuous shaking. Samples were read at 600 nm after 48 h of incubation. Ceftriaxone was used as a positive control. Experiments were carried out in triplicate. The minimum inhibitory concentration (MIC) is defined as the lowest concentration of an antimicrobial agent that inhibits visible growth of a microorganism. MIC values were generated from non-linear fitted curves (growth inhibition versus log of concentration) using Equation (2) (Gompertz function) [83].

(2) $$\mathrm{MIC}=10^{\left(M+\frac{1}{B}\right)}$$

where M is the log concentration of the inflection point and B is a slope parameter.

### 3.13. Wound-Healing Effect of E. hyemale Extract in Type 2 Diabetic Rats 

The present protocol was reviewed and approved by the Ethics Committee of the Autonomous University of Coahuila (TD-01-09-21-1). Rats were obtained from the biotherium Morelos. All animals received appropriate care according to the animal welfare guidelines established by the Mexican regulation NOM-062-ZOO-1999 and the Guide for the Care and Use of Laboratory Animals in the USA. Rats were housed in a temperature- and humidity-controlled rooms in our animal facilities with a 12 h light/dark cycle. Rats were allowed to acclimate for one week prior to the start of the study. Thirty male Wistar rats weighing 250–300 g (10–12 weeks old) were used. Throughout the study, rats had free access to standard rat chow and tap water ad libitum.

Type 2 diabetes was induced by a single intraperitoneal (i.p.) injection of nicotinamide (150 mg/kg). Fifteen minutes later, the animals received an injection of streptozotocin (STZ; 65 mg/kg, i.p.), as reported [84]. STZ was dissolved in 0.1 M citrate buffer (pH 4). Only rats with glucose levels above 200 mg/dL were included. Glucose levels were measured using a glucometer (Accu-Chek) and a drop of blood from the rats’ tail. 

The diabetic rats were anesthetized with pentobarbital (60 mg/kg, i.p.). After anesthesia, the back of the rats was shaved with an electric clipper. A round wound was then made on the back of each rat using a sterilized 10 mm biopsy punch. The rats were randomly divided into three groups: (1) non-diabetic control group (n = 6), injected with citrate buffer only; (2) type 2 diabetic control group (n = 6), both treated daily with physiological saline (0.9% v/v); and (3) type 2 diabetic groups treated daily with the extract (0.6, 1.2, and 2.4 mg/mL, n = 6 each). Wound size was recorded by camera and caliper at 0, 3, 7, 11, 14, 17, and 21 days. The percentage of wound contraction was calculated from the initial wound size using Equation (3):

(3) $$\%\;\mathrm{wound}\;\mathrm{contraction}=\frac{\mathrm{Initial}\;\mathrm{wound}\;\mathrm{area}-\mathrm{specific}\;\mathrm{day}\;\mathrm{wound}\;\mathrm{area}\;}{\mathrm{Initial}\;\mathrm{wound}\;\mathrm{area}}\times 100$$

### 3.14. Histological Analysis

Complete healing was observed at 21, 28, 24, 14, and 21 days in non-diabetic rats, type 2 diabetic rats, and type 2 diabetic rats treated with the extract (0.6, 1.2, and 2.4 mg/mL), respectively. The rats (n = 6 per group) were then anesthetized with pentobarbital (60 mg/kg, i.p.) and the healed dorsal skin was removed and placed in neutral buffered 4% v/v formalin for 24 h. Skin samples were dehydrated in ethanol and embedded in paraffin. Paraffin blocks were sectioned on a rotary microtome to obtain 5 µm skin cross sections and stained with Masson’s trichrome kit according to the supplier’s recommendation (Hycel, Guadalajara, JAL, Mexico) to measure collagen deposition. Tissue visualization was performed using an optical microscope. Collagen deposition was quantified using ImageJ software [85].

### 3.15. Statistical Analysis

Phenolic and flavonoid concentrations are expressed as mean ± standard deviation, whereas biological data are expressed as mean ± standard error of the mean (SEM). Differences in cell viability, proliferation, cytokine release, and collagen deposition were evaluated using the Tukey test after one-way or two-way analysis of variance (ANOVA) of repeated measures (wound healing data) was performed. Statistical significance was accepted at *p* < 0.05. All statistical analyses were performed with GraphPad Prism v8.0.

## 4. Conclusions

Ultrasound- and microwave-assisted extraction can be used to obtain minerals such as potassium, calcium, and silicon; sterols such as campesterol, γ-sitosterol, and cycloartenol; phenolic acids such as caffeic acid 4-O-glucoside and ferulic acid 4-O-glucoside; flavonols such as kaempferol 3,7,4’-O-triglucoside, kaempferol 3,7-O-diglucoside, and quercetin; a phenylpropanoid such as phenol-2,4-bis(1-phenylethyl); and a lignan such as conidendrin from the Mexican *E. hyemale* plant. This is the first time that both phenol-2,4-bis(1-phenylethyl) and conidendrin have been identified in this plant. The extract obtained by this technique has a crystalline structure with a high presence of polar groups and shows resistance to thermal degradation. Its pharmacological properties include antimicrobial effects against *S. aureus* and *E. coli*, control of inflammation by increasing IL-10 and inhibiting MCP-1 released by macrophages, and promotion of collagen synthesis by dermal fibroblasts. It is highly likely that its anti-inflammatory and collagen-synthesizing effects benefited to faster wound healing in diabetic rats.

## Figures and Tables

**Table 1:** Chemical elements found in *E. hyemale* extract by X-ray fluorescence. Potassium (K), calcium (Ca), silicon (Si), chlorine (Cl), sulfur (S), phosphorus (P), iron (Fe), and magnesium (Mg).

Chemical Element	Concentration (%)	Concentration Based on Ash (%)
K	40.00	5.72
Ca	23.97	3.42
Si	23.20	3.31
Cl	3.82	0.54
S	3.42	0.48
P	2.73	0.39
Fe	1.09	0.15
Mg	0.90	0.13

**Table 2:** Components identified by gas chromatography (CG-MS) in the extract of *E. hyemale*. Rt, retention time; m/z, mass-to-charge ratio; min, minutes.

Rt (min)	m/z [M-H]^−^	Identified Component	Family	Structure
16.44	315.2	Campesterol	Phytosterols	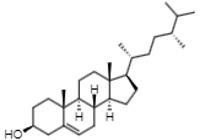
18.89	329.2	γ-sitosterol	Phytosterols	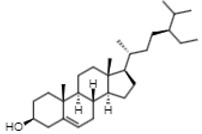
17.30	287.2	Phenol, 2,4-bis(1-phenylethyl)	Phenylpropenoids	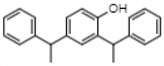
20.80	41	Cycloartenol	Phytosterols	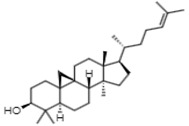

**Table 3:** Components identified by reverse-phase high-performance liquid chromatography (RP-HPLC-ESI-MS) in the extract of *E. hyemale*. Rt, retention time; m/z, mass-to-charge ratio; min, minutes.

Rt (min)	m/z [M-H]^−^	Identified Component	Family	Structure
8.67	340.9	Caffeic acid 4-O-glucoside	Hydroxycinnamic acids	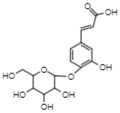
29.06	770.8	Kaempferol 3,7,4’-O-triglucoside	Flavonols	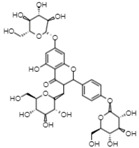
31.16	355	Ferulic acid 4-O-glucoside	Methoxycinnamic acids	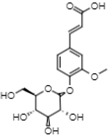
35.52	355	Conidendrin	Lignans	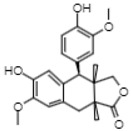
39.6	300.9	Quercetin	Flavonols	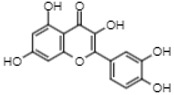
42.17	609	Kaempferol 3,7-O-diglucoside	Flavonols	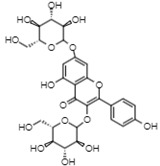

## Data Availability

The data presented in this study are available upon request from the corresponding author.

## References

[1] Pryer K.M., Tomasi C., Wang X., Meineke E.K., Windham M.D. (2020). Using computer vision on herbarium specimen images to discriminate among closely related horsetails (Equisetum). App. Plant Sci..

[2] Gallardo-Pérez J.C., Esparza-Aguilar M.d.L., Gómez-Campos A. (2006). Importancia etnobotánica de una planta vascular sin semilla en México: Equisetum. Polibotánica.

[3] Argueta A. (1994). Atlas de las Plantas de la Medicina Tradicional Mexicana.

[4] Perez Gutierrez R.M., Laguna G.Y., Walkowski A. (1985). Diuretic activity of Mexican equisetum. J. Ethnopharmacol..

[5] De Queiroz L.N., Da Fonseca A.C.C., Wermelinger G.F., da Silva D.P.D., Pascoal A.C.R.F., Sawaya A.C.H.F., de Almeida E.C.P., do Amaral B.S., de Lima Moreira D., Robbs B.K. (2023). New substances of *Equisetum hyemale* L. extracts and their in vivo antitumoral effect against oral squamous cell carcinoma. J. Ethnopharmacol..

[6] Carmignan F., Matias R., Carollo C.A., Dourado D.M., Fermiano M.H., Silva B.A.K., Bastos P. (2020). Efficacy of application of Equisetum pyramidale Goldm. hydrogel for tissue restoration of induced skin lesions in Wistar rats. Braz. J. Biol..

[7] De Queiroz G.M., Politi F.A., Rodrigues E.R., Souza-Moreira T.M., Moreira R.R., Cardoso C.R., Santos L.C., Pietro R.C. (2015). Phytochemical Characterization, Antimicrobial Activity, and Antioxidant Potential of *Equisetum hyemale* L. (Equisetaceae) Extracts. J. Med. Food.

[8] Dos Santos Alves C.F., Bonez P.C., de Souza M.E., Casagrande C., Freitas L., Dolwitsch C., Pires F., Rorato Sagrillo M., Fernandes de Brum G., Anraku de Campos M.M. (2019). Antimicrobial, Cyto and Genotoxic Activities of *Equisetum hyemale*. Pharmacogn. J..

[9] Dos Santos Alves C.F., Bonez P.C., de Souza M.E., da Cruz R.C., Boligon A.A., Piana M., Brum T.F., Rossi G.G., Jesus R.D., Grando T.H. (2016). Antimicrobial, antitrypanosomal and antibiofilm activity of *Equisetum hyemale*. Microb. Pathog..

[10] Li Q., Li X., Zheng B., Zhao C. (2021). The optimization of ultrasonic-microwave assisted synergistic extraction of Lotus plumule extract rich in flavonoids and its hypoglycemic activity. Food Prod. Process. Nutr..

[11] Carneiro D.M., Freire R.C., Honorio T.C., Zoghaib I., Cardoso F.F., Tresvenzol L.M., de Paula J.R., Sousa A.L., Jardim P.C., da Cunha L.C. (2014). Randomized, Double-Blind Clinical Trial to Assess the Acute Diuretic Effect of *Equisetum arvense* (Field Horsetail) in Healthy Volunteers. Evid. Based Complement. Alternat. Med..

[12] Grundemann C., Lengen K., Sauer B., Garcia-Kaufer M., Zehl M., Huber R. (2014). *Equisetum arvense* (common horsetail) modulates the function of inflammatory immunocompetent cells. BMC Complement. Altern. Med..

[13] Parrish A.N., Lange I., Samec D., Lange B.M. (2022). Differential Accumulation of Metabolites and Transcripts Related to Flavonoid, Styrylpyrone, and Galactolipid Biosynthesis in Equisetum Species and Tissue Types. Metabolites.

[14] Ozay Y., Kasim Cayci M., Guzel-Ozay S., Cimbiz A., Gurlek-Olgun E., Sabri Ozyurt M. (2013). Effects of *Equisetum arvense* Ointment on Diabetic Wound Healing in Rats. Wounds.

[15] Singh S., Young A., McNaught C.-E. (2017). The physiology of wound healing. Surgery.

[16] Gianino E., Miller C., Gilmore J. (2018). Smart Wound Dressings for Diabetic Chronic Wounds. Bioengineering.

[17] Han G., Ceilley R. (2017). Chronic Wound Healing: A Review of Current Management and Treatments. Adv. Ther..

[18] Bowler P.G., Duerden B.I., Armstrong D.G. (2001). Wound microbiology and associated approaches to wound management. Clin. Microbiol. Rev..

[19] Laires M.J., Monteiro C. (2008). Exercise, magnesium and immune function. Magnes. Res..

[20] Volpe S.L. (2013). Magnesium in Disease Prevention and Overall Health. Adv. Nutr..

[21] Ward R.J., Crichton R.R., Taylor D.L., Della Corte L., Srai S.K., Dexter D.T. (2011). Iron and the immune system. J. Neural Transm..

[22] Wang N., Ma Y., Shi H., Song Y., Guo S., Yang S. (2022). Mg-, Zn-, and Fe-Based Alloys with Antibacterial Properties as Orthopedic Implant Materials. Front. Bioeng. Biotechnol..

[23] Blanco I., Siracusa V. (2021). The Use of Thermal Techniques in the Characterization of Bio-Sourced Polymers. Materials.

[24] Leyva-Porras C., Cruz-Alcantar P., Espinosa-Solis V., Martinez-Guerra E., Balderrama C.I.P., Martinez I.C., Saavedra-Leos M.Z. (2019). Application of Differential Scanning Calorimetry (DSC) and Modulated Differential Scanning Calorimetry (MDSC) in Food and Drug Industries. Polymers.

[25] De Assis A.C.L., Alves L.P., Malheiro J.P.T., Barros A.R.A., Pinheiro-Santos E.E., de Azevedo E.P., Silva Alves H.D., Oshiro-Junior J.A., Damasceno B. (2019). *Opuntia Ficus-Indica*, L. Miller (*Palma forrageira*) as an Alternative Source of Cellulose for Production of Pharmaceutical Dosage Forms and Biomaterials: Extraction and Characterization. Polymers.

[26] Masłowski M., Miedzianowska J., Czylkowska A., Strzelec K. (2020). Horsetail (*Equisetum arvense*) as a Functional Filler for Natural Rubber Biocomposites. Materials.

[27] Gierlinger N., Sapei L., Paris O. (2008). Insights into the chemical composition of *Equisetum hyemale* by high resolution Raman imaging. Planta.

[28] Joshi D.D., Joshi D.D. (2012). FTIR Spectroscopy: Herbal Drugs and Fingerprints. Herbal Drugs and Fingerprints: Evidence Based Herbal Drugs.

[29] Durak T., Depciuch J. (2020). Effect of plant sample preparation and measuring methods on ATR-FTIR spectra results. Environ. Exp. Bot..

[30] Milutinović M., Radovanović N., Rajilić-Stojanović M., Šiler-Marinković S., Dimitrijević S., Dimitrijević-Branković S. (2014). Microwave-assisted extraction for the recovery of antioxidants from waste *Equisetum arvense*. Ind. Crops Prod..

[31] Do Monte F.H., dos Santos J.G., Russi M., Lanziotti V.M., Leal L.K., Cunha G.M. (2004). Antinociceptive and anti-inflammatory properties of the hydroalcoholic extract of stems from *Equisetum arvense* L. in mice. Pharmacol. Res..

[32] Gomez M.A., Saenz M.T., Garcia M.D., Fernandez M.A. (1999). Study of the topical anti-inflammatory activity of Achillea ageratum on chronic and acute inflammation models. Z. Naturforsch. C J. Biosci..

[33] Navarro A., De las Heras B., Villar A. (2001). Anti-inflammatory and immunomodulating properties of a sterol fraction from Sideritis foetens Clem. Biol. Pharm. Bull..

[34] PubChem Antiinflammatory Activity in Human Neutrophils Assessed as fMLP-Induced Superoxide Release after 5 min by Spectrometry. https://pubchem.ncbi.nlm.nih.gov/bioassay/311335.

[35] Chen J.J., Chen P.H., Liao C.H., Huang S.Y., Chen I.S. (2007). New phenylpropenoids, bis(1-phenylethyl)phenols, bisquinolinone alkaloid, and anti-inflammatory constituents from *Zanthoxylum integrifoliolum*. J. Nat. Prod..

[36] Niu H., Li X., Yang A., Jin Z., Wang X., Wang Q., Yu C., Wei Z., Dou C. (2018). Cycloartenol exerts anti-proliferative effects on Glioma U87 cells via induction of cell cycle arrest and p38 MAPK-mediated apoptosis. J. BUON.

[37] Zhang Z.L., Luo Z.L., Shi H.W., Zhang L.X., Ma X.J. (2017). Research advance of functional plant pharmaceutical cycloartenol about pharmacological and physiological activity. Zhongguo Zhong Yao Za Zhi.

[38] Thuluva S.C., Igel M., Giesa U., Lutjohann D., Sudhop T., von Bergmann K. (2005). Ratio of lathosterol to campesterol in serum predicts the cholesterol-lowering effect of sitostanol-supplemented margarine. Int. J. Clin. Pharmacol. Ther..

[39] Fons F., Froissard D., Bessière J.-M., Fruchier A., Buatois B., Rapior S. (2013). Volatile Composition of Six Horsetails: Prospects and Perspectives. Nat. Prod. Commun..

[40] Giordani C., Waller S.B., Madrid I.M., Guterres K.A., de Matos C.B., Hoffmann J.F., de Castro L.L., Chaves F.C., de Faria R.O., Cleff M.B. (2022). Chemical, antioxidant and cytotoxic profile of hydroalcoholic extracts of plants from Southern Brazil and their activity against pathogenic fungi isolated from dogs and cats with sensitivity and resistance to conventional antifungals. Nat. Prod. Res..

[41] Li H., Wang P., Liu Q., Cheng X., Zhou Y., Xiao Y. (2012). Cell cycle arrest and cell apoptosis induced by *Equisetum hyemale* extract in murine leukemia L1210 cells. J. Ethnopharmacol..

[42] Riss T.L., Moravec R.A., Niles A.L., Duellman S., Benink H.A., Worzella T.J., Minor L., Markossian S., Grossman A., Brimacombe K., Arkin M., Auld D., Austin C., Baell J., Chung T.D.Y., Coussens N.P., Dahlin J.L. (2004). Cell Viability Assays. Assay Guidance Manual.

[43] Keith C.T., Borisy A.A., Stockwell B.R. (2005). Multicomponent therapeutics for networked systems. Nat. Rev. Drug Discov..

[44] Sundarraj S., Thangam R., Sreevani V., Kaveri K., Gunasekaran P., Achiraman S., Kannan S. (2012). γ-Sitosterol from Acacia nilotica L. induces G2/M cell cycle arrest and apoptosis through c-Myc suppression in MCF-7 and A549 cells. J. Ethnopharmacol..

[45] Rajendra Prasad N., Karthikeyan A., Karthikeyan S., Reddy B.V. (2011). Inhibitory effect of caffeic acid on cancer cell proliferation by oxidative mechanism in human HT-1080 fibrosarcoma cell line. Mol. Cell. Biochem..

[46] Li H., Yang L., Zhang Y., Gao Z. (2016). Kaempferol inhibits fibroblast collagen synthesis, proliferation and activation in hypertrophic scar via targeting TGF-β receptor type I. Biomed. Pharmacother..

[47] Lampiasi N., Montana G. (2016). The molecular events behind ferulic acid mediated modulation of IL-6 expression in LPS-activated Raw 264.7 cells. Immunobiology.

[48] Mi Y., Zhong L., Lu S., Hu P., Pan Y., Ma X., Yan B., Wei Z., Yang G. (2022). Quercetin promotes cutaneous wound healing in mice through Wnt/β-catenin signaling pathway. J. Ethnopharmacol..

[49] Nair A.N.S., Nair R.V.R., Nair A.P.R., Nair A.S., Thyagarajan S., Johnson A.J., Baby S. (2020). Antidiabetes constituents, cycloartenol and 24-methylenecycloartanol, from Ficus krishnae. PLoS ONE.

[50] Abate G., Zhang L., Pucci M., Morbini G., Mac Sweeney E., Maccarinelli G., Ribaudo G., Gianoncelli A., Uberti D., Memo M. (2021). Phytochemical Analysis and Anti-Inflammatory Activity of Different Ethanolic Phyto-Extracts of *Artemisia annua* L.. Biomolecules.

[51] Yuan L., Zhang F., Shen M., Jia S., Xie J. (2019). Phytosterols Suppress Phagocytosis and Inhibit Inflammatory Mediators via ERK Pathway on LPS-Triggered Inflammatory Responses in RAW264.7 Macrophages and the Correlation with Their Structure. Foods.

[52] Singampalli K.L., Balaji S., Wang X., Parikh U.M., Kaul A., Gilley J., Birla R.K., Bollyky P.L., Keswani S.G. (2020). The Role of an IL-10/Hyaluronan Axis in Dermal Wound Healing. Front. Cell. Dev. Biol..

[53] Leyva-López N., Gutierrez-Grijalva E.P., Ambriz-Perez D.L., Heredia J.B. (2016). Flavonoids as Cytokine Modulators: A Possible Therapy for Inflammation-Related Diseases. Int. J. Mol. Sci..

[54] Sabeva N.S., McPhaul C.M., Li X., Cory T.J., Feola D.J., Graf G.A. (2011). Phytosterols differentially influence ABC transporter expression, cholesterol efflux and inflammatory cytokine secretion in macrophage foam cells. J. Nutr. Biochem..

[55] Aherne S.A., O’Brien N.M. (2008). Modulation of cytokine production by plant sterols in stimulated human Jurkat T cells. Mol. Nutr. Food Res..

[56] Sun S.J., Yu W.Q., Zhang Y.L., Jiang X.Q., Zhang F.Q. (2013). Effects of TiO2 nanotube layers on RAW 264.7 macrophage behaviour and bone morphogenetic protein-2 expression. Cell Prolifer..

[57] Chang C.F., Liao K.C., Chen C.H. (2017). 2-Phenylnaphthalene Derivatives Inhibit Lipopolysaccharide-Induced Pro-Inflammatory Mediators by Downregulating of MAPK/NF-kappaB Pathways in RAW 264.7 Macrophage Cells. PLoS ONE.

[58] Huang C., Li W., Zhang Q., Chen L., Chen W., Zhang H., Ni Y. (2018). Anti-inflammatory activities of Guang-Pheretima extract in lipopolysaccharide-stimulated RAW 264.7 murine macrophages. BMC Complement. Altern. Med..

[59] Cantuária A.P.C., Figueiredo T.M., Freire M.S., Lima S.M.F., Almeida J.A., Franco O.L., Rezende T.M.B. (2018). The effects of glucose concentrations associated with lipopolysaccharide and interferon-gamma stimulus on mediators’ production of RAW 264.7 cells. Cytokine.

[60] Astashkina A., Mann B., Grainger D.W. (2012). A critical evaluation of in vitro cell culture models for high-throughput drug screening and toxicity. Pharmacol. Ther..

[61] Brown T.D. (2000). Techniques for mechanical stimulation of cells in vitro: A review. J. Biomech..

[62] Búfalo M.C., Ferreira I., Costa G., Francisco V., Liberal J., Cruz M.T., Lopes M.C., Batista M.T., Sforcin J.M. (2013). Propolis and its constituent caffeic acid suppress LPS-stimulated pro-inflammatory response by blocking NF-κB and MAPK activation in macrophages. J. Ethnopharmacol..

[63] Endale M., Park S.-C., Kim S., Kim S.-H., Yang Y., Cho J.Y., Rhee M.H. (2013). Quercetin disrupts tyrosine-phosphorylated phosphatidylinositol 3-kinase and myeloid differentiation factor-88 association, and inhibits MAPK/AP-1 and IKK/NF-κB-induced inflammatory mediators production in RAW 264.7 cells. Immunobiology.

[64] Bak M.-J., Hong S.-G., Lee J.-W., Jeong W.-S. (2012). Red Ginseng Marc Oil Inhibits iNOS and COX-2 via NFκB and p38 Pathways in LPS-Stimulated RAW 264.7 Macrophages. Molecules.

[65] Palacz-Wrobel M., Borkowska P., Paul-Samojedny M., Kowalczyk M., Fila-Danilow A., Suchanek-Raif R., Kowalski J. (2017). Effect of apigenin, kaempferol and resveratrol on the gene expression and protein secretion of tumor necrosis factor alpha (TNF-α) and interleukin-10 (IL-10) in RAW-264.7 macrophages. Biomed. Pharmacother..

[66] European Committee for Antimicrobial Susceptibility Testing (EUCAST) of the European Society of Clinical Microbiology and Infectious Diseases (2003). Determination of minimum inhibitory concentrations (MICs) of antibacterial agents by broth dilution. Clin. Microbiol. Infect..

[67] Čanadanović-Brunet J.M., Ćetković G.S., Djilas S.M., Tumbas V.T., Savatović S.S., Mandić A.I., Markov S.L., Cvetković D.D. (2009). Radical scavenging and antimicrobial activity of horsetail (*Equisetum arvense* L.) extracts. Int. J. Food Sci. Technol..

[68] Xie Y., Yang W., Tang F., Chen X., Ren L. (2015). Antibacterial activities of flavonoids: Structure-activity relationship and mechanism. Curr. Med. Chem..

[69] McRae J., Yang Q., Crawford R., Palombo E. (2007). Review of the methods used for isolating pharmaceutical lead compounds from traditional medicinal plants. Environmentalist.

[70] Khameneh B., Iranshahy M., Soheili V., Fazly Bazzaz B.S. (2019). Review on plant antimicrobials: A mechanistic viewpoint. Antimicrob. Resist. Infect. Control.

[71] Kenawy E.-R., Worley S.D., Broughton R. (2007). The Chemistry and Applications of Antimicrobial Polymers:  A State-of-the-Art Review. Biomacromolecules.

[72] Calabrese E.J., Baldwin L.A. (2001). U-shaped dose-responses in biology, toxicology, and public health. Annu. Rev. Public Health.

[73] Karim S., Alkreathy H.M., Ahmad A., Khan M.I. (2021). Effects of Methanolic Extract Based-Gel from Saudi Pomegranate Peels with Enhanced Healing Potential on Excision Wounds in Diabetic Rats. Front. Pharmacol..

[74] Nagar H.K., Srivastava A.K., Srivastava R., Kurmi M.L., Chandel H.S., Ranawat M.S. (2016). Pharmacological Investigation of the Wound Healing Activity of *Cestrum nocturnum* (L.) Ointment in Wistar Albino Rats. J. Pharm..

[75] Lodhi S., Singhai A.K. (2013). Wound healing effect of flavonoid rich fraction and luteolin isolated from *Martynia annua* Linn. on streptozotocin induced diabetic rats. Asian Pac. J. Trop. Med..

[76] Aly S.H., El-Hassab M.A., Elhady S.S., Gad H.A. (2023). Comparative Metabolic Study of *Tamarindus indica* L.’s Various Organs Based on GC/MS Analysis, In Silico and In Vitro Anti-Inflammatory and Wound Healing Activities. Plants.

[77] Priyadarshini C.F. (2022). Comparative study of hydroalcoholic extracts of Bryophyllum pinnatum and Macrotyloma uniflorum for their antioxidant, antiurolithiatic, and wound healing potential. J. Appl. Biol. Biotechnol..

[78] Mssillou I., Agour A., Slighoua M., Chebaibi M., Amrati F.E., Alshawwa S.Z., Kamaly O.A., El Moussaoui A., Lyoussi B., Derwich E. (2022). Ointment-Based Combination of *Dittrichia viscosa* L. and *Marrubium vulgare* L. Accelerate Burn Wound Healing. Pharmaceuticals.

[79] Carvalho M.T.B., Araújo-Filho H.G., Barreto A.S., Quintans-Júnior L.J., Quintans J.S.S., Barreto R.S.S. (2021). Wound healing properties of flavonoids: A systematic review highlighting the mechanisms of action. Phytomedicine.

[80] Vitale S., Colanero S., Placidi M., Di Emidio G., Tatone C., Amicarelli F., D’Alessandro A.M. (2022). Phytochemistry and Biological Activity of Medicinal Plants in Wound Healing: An Overview of Current Research. Molecules.

[81] Ezzat S.M., Choucry M.A., Kandil Z.A. (2016). Antibacterial, antioxidant, and topical anti-inflammatory activities of Bergia ammannioides: A wound-healing plant. Pharm. Biol..

[82] Solís-Salas L.M., Sierra-Rivera C.A., Cobos-Puc L.E., Ascacio-Valdés J.A., Silva-Belmares S.Y. (2021). Antibacterial Potential by Rupture Membrane and Antioxidant Capacity of Purified Phenolic Fractions of *Persea americana* Leaf Extract. Antibiotics.

[83] Lambert R.J.W., Pearson J. (2000). Susceptibility testing: Accurate and reproducible minimum inhibitory concentration (MIC) and non-inhibitory concentration (NIC) values. J. Appl. Microbiol..

[84] Ghasemi A., Khalifi S., Jedi S. (2014). Streptozotocin-nicotinamide-induced rat model of type 2 diabetes (review). Acta Physiol. Hung..

[85] Aceros H., Farah G., Cobos-Puc L., Stabile A.M., Noiseux N., Mukaddam-Daher S. (2011). Moxonidine improves cardiac structure and performance in SHR through inhibition of cytokines, p38 MAPK and Akt. Br. J. Pharmacol..

